# *Larimichthys crocea* Swim Bladder Polysaccharides Attenuate 5-Fluorouracil-Induced Intestinal Injury by Modulating the Gut–Metabolic Axis

**DOI:** 10.3390/foods15081425

**Published:** 2026-04-19

**Authors:** Shouhao Zhao, Ruixue Zhao, Donglin Sui, Yixuan Li, Huan Li, Shugang Li, Chunqing Ai, Xueting Bai, Yilin Sha, Jingxian Yan, Wudeng Wang, Xiaomeng Ren

**Affiliations:** 1 State Key Laboratory of Marine Food Processing and Safety Control, National Engineering Research Center of Seafood, School of Food Science and Technology, Dalian Polytechnic University, Dalian 116034, China; shouhao1105@163.com (S.Z.);; 2 Research Institute of Photonics, Dalian Polytechnic University, Dalian 116034, China

**Keywords:** *Larimichthys crocea*, swim bladder polysaccharides, 5-Fluorouracil, intestinal injury, gut microbiota, metabolic regulation

## Abstract

5-Fluorouracil (5-FU) is a first-line chemotherapeutic agent for solid tumors, but its clinical application is severely limited by dose-dependent intestinal injury that impairs patient quality of life and compromises therapeutic efficacy. Natural polysaccharides, especially marine-derived ones, have become safe and multi-targeted gut-protective candidates due to their excellent biocompatibility and prebiotic-like activities. *Larimichthys crocea* swim bladder is a characteristic marine biological resource, and its polysaccharides (CIPs) have shown potential bioactivities, yet their protective mechanism against 5-FU-induced intestinal injury remains unclear. Our study explored the protective effects of *Larimichthys crocea* swim bladder polysaccharides (CIPs) against 5-FU-induced intestinal injury in mice. Following 14-day preventive administration, CIPs alleviated 5-FU-induced body weight loss, diarrhea, colonic shortening, and mucosal injury, and restored goblet cell function. Mechanistically, CIPs enhanced intestinal barrier integrity by upregulating ZO-1, Occludin, and MUC2, suppressed the MyD88/NF-κB pathway to balance inflammatory cytokines, and ameliorated oxidative stress by regulating MDA, GSH, SOD, and CAT. CIPs also restored gut microbial diversity and the Firmicutes/Bacteroidota ratio, and modulated retinol and arginine metabolism. In vitro, CIPs reduced inflammation and oxidative damage in Caco-2 cells and promoted M2 macrophage polarization. Thus, CIPs alleviate 5-FU-induced intestinal injury via multi-targeted regulation of the gut–metabolic axis, showing great potential as a dietary intervention and gut health support agent in food science and oncology nutrition, and boosting the high-value utilization of marine resources.

## 1. Introduction

Chemotherapy serves as the cornerstone of clinical management for multiple solid malignancies; however, chemotherapy-induced intestinal injury represents a prevalent and severe dose-limiting toxicity that significantly compromises treatment adherence and patient clinical outcomes. As a fundamental chemotherapeutic agent, 5-Fluorouracil (5-FU) is widely utilized for the treatment of colorectal cancer, breast cancer, and various other cancers, but its clinical application is restricted by gastrointestinal toxicity, including mucositis, diarrhea, abdominal pain, and barrier dysfunction [1,2]. These adverse effects markedly impair patient quality of life and often necessitate dose reduction or discontinuation, ultimately compromising therapeutic efficacy [3]. Nutritional supplementation, a key component of current supportive strategies, is often inadequate and may cause additional side effects [4].

The development of intestinal injury following 5-FU administration arises from a multifactorial pathogenesis, primarily involving compromised intestinal barrier function, sustained inflammatory activation, and disruption of microbial homeostasis. 5-FU damages epithelial cells and tight junction proteins, increases intestinal permeability, and induces the release of pro-inflammatory cytokines [5,6]. It also perturbs gut microbiota homeostasis, reducing probiotics such as *Bifidobacteria* and *Lactobacilli* and short-chain fatty acids (SCFAs) while favoring pathogenic overgrowth, which further exacerbates mucosal injury [7,8].

Natural polysaccharides have garnered interest as safe, multi-target modulators of intestinal health. These substances are poorly absorbed by the host but undergo fermentation by gut microbiota to generate SCFAs, which exert anti-inflammatory, barrier-protective, and regulation of immune responses [9,10]. Investigations have demonstrated that okra polysaccharides mend the mucosal barrier through the regulation of gut microbiota and the IL-10 pathway [5]; additionally, they alleviate chemotherapy-triggered intestinal mucosal inflammation through targeting gut microbiota and linoleic acid turnover [11].

Swim bladders contain proteins, polysaccharides, and trace elements with diverse bioactivities [12,13]. Polysaccharides from the *Larimichthys crocea* swim bladder. The *Larimichthys crocea* is a vital marine fish in China, possessing antioxidant, anti-inflammatory, hepatoprotective, and wound-healing properties [14,15,16,17]. However, while CIPs have been reported to exhibit protective effects in colitis models [18], their protective efficacy and underlying mechanisms against 5-FU-induced intestinal injury have never been investigated, representing a critical research gap. We hypothesized that CIPs attenuate 5-FU-induced intestinal injury via regulating the gut–metabolic axis, and this study aimed to investigate the protective effects and mechanisms of CIPs against 5-FU-triggered intestinal toxicity, focusing on intestinal barrier integrity, inflammatory response, and gut microbiota homeostasis. Collectively, these findings are expected to position CIPs as promising candidates for functional food development in oncology nutrition.

## 2. Materials and Methods

### 2.1. Reagents

5-Fluorouracil (5-FU) was supplied by Yuanye Biotech Co., Ltd. (Shanghai, China). An extra-ultrasensitive ECL chemiluminescent substrate kit was purchased from Beyotime Biotechnology Co., Ltd. (Shanghai, China). Antibodies against p-NF-κB, NF-κB, IκBα, and p-IκBα were acquired from ABclonal (Wuhan, China). Antibodies for MUC2, ZO-1, Occludin, Claudin-1, BAX, MyD88, β-actin, and GAPDH were provided by Proteintech (Wuhan, China). Assay kits for GSH, MDA, SOD, and CAT were obtained from Nanjing Jiancheng Biotechnology Co., Ltd. (Nanjing, China).

### 2.2. Extraction of CIPs

**Figure 1:**
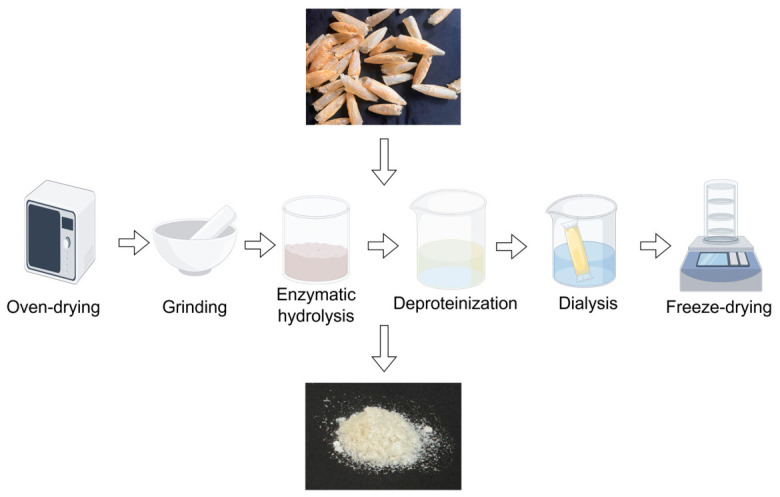
Preparation of a homogenous polysaccharide CIPs from *Larimichthys crocea* polysaccharides.

CIPs were prepared based on our team’s previous established protocol with detailed supplementary procedures [19]. Extraction procedures are shown in Figure 1. Briefly, the raw *Larimichthys crocea* swim bladder was used as the starting material. Polysaccharides were extracted by enzymatic hydrolysis at 37 °C for 2 h. The Sevag reagent method was applied to remove protein from the crude extract. The deproteinized fraction was dialyzed in a 3500 Da dialysis bag, and the purified retentate was lyophilized to obtain CIPs.

### 2.3. Animal Experiments

Forty healthy male C57BL/6 mice (6 weeks old, average weight 20 ± 2 g) were purchased from Liaoning Changsheng Biotechnology Co., Ltd. (Benxi, China). Mice were maintained in an individually ventilated cage (IVC) system(Beijing HFK Bioscience Co., Ltd., Beijing, China) under controlled environmental conditions (temperature: 22 ± 2 °C, relative humidity: 50% ± 5%, 12 h light/dark cycle) with free access to sterile water and food. All animal experimental protocols were reviewed and approved by the Institutional Animal Care and Use Committee (IACUC) of Dalian Polytechnic University (Approval No.: DLPU2025048, approved on 3 May 2025).

**Figure 2:**
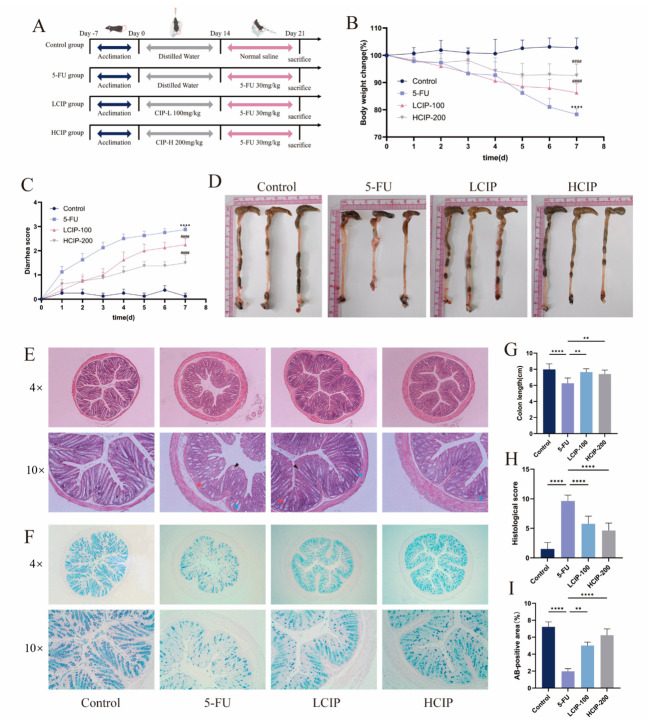
CIPs ameliorate 5-FU-induced intestinal injury in mice. (**A**) Schematic diagram of the animal experimental protocol. (**B**) Changes in body weight of mice during the experimental period. (**C**) Diarrhea score change. (**D**,**G**) Representative images and quantification of colon length in each group. (**E**) Hematoxylin and eosin (H&E) staining of colon tissues showing mucosal morphology. Arrowhaed inflammatory cell infiltrates within mucosa (red) and submucosa (blue); black arrowhead, goblet cell loss. (**F**) Alcian blue-periodic acid-Schiff (AB-PAS) staining of colon tissues. (**H**,**I**) Histopathological scores and quantitative analysis of colon tissue damage. Data are presented as mean ± SEM (*n* = 8). ** *p* < 0.01, and **** *p* < 0.0001 vs. the 5-FU group; ^####^ *p* < 0.0001 vs. the control group.

Mice were acclimatized for 1 week before experimentation, with a total experimental period of 21 days. The study protocol is depicted in Figure 2A. After a 7-day acclimation (Day −7 to 0), 40 mice were randomly divided into four groups (*n* = 10 each) and underwent a 21-day experiment (Day 0–21, Figure 2A). The control group received daily oral gavage of distilled water (Day 0–14) followed by intraperitoneal (i.p.) normal saline (Day 14–21). The 5-FU model group received oral distilled water (Day 0–14), then i.p. 30 mg/kg/day 5-FU (Day 14–21). The LCIP and HCIP groups received 100 or 200 mg/kg/day CIPs, respectively, via oral gavage on Day 0–14, followed by 30 mg/kg/day 5-FU on Day 14–21. Doses were selected based on our previous work [18]. All oral doses were given in 200 μL from Day 0. Mice had free access to sterile water until euthanasia on Day 21.

Throughout the study, animals’ body weight was measured and recorded daily, and their fecal traits as well as diarrhea conditions were also monitored. Diarrhea was scored based on a validated scoring method.

On Day 21, mice were anesthetized via intraperitoneal injection of 1% pentobarbital sodium at 50 mg/kg body weight. Blood samples were collected from the retro-orbital plexus under sterile conditions. Whole blood was left to clot at room temperature for 30 min, then centrifuged at 3000× *g* for 15 min at 4 °C to isolate serum, which was stored at −80 °C until analysis.

Following blood collection, mice were euthanized by cervical dislocation. The jejunum was rapidly dissected: a 1 cm segment was fixed in 4% paraformaldehyde for 24 h for histological examination, and the remaining tissue was snap-frozen in liquid nitrogen and stored at −80 °C for subsequent RT-qPCR and Western blot analyses.

### 2.4. Reagent Kit Testing

Serum was isolated by centrifugation at 1000× *g* for 10 min at 4 °C, and serum levels of oxidative stress-related indicators (MDA, GSH, SOD, and CAT) were measured using commercially available assay kits following the manufacturers’ instructions, as 5-FU-induced intestinal injury elicits systemic redox imbalance and serum reflects the overall oxidative status [20,21].

### 2.5. Histological Analysis

Distal colonic tissues were fixed in 4% paraformaldehyde for 24 h, embedded in paraffin, and sectioned at 5 μm thickness. Following a previously described protocol [22] with minor adjustments, tissue sections were stained with H&E and AB-PAS. Morphological features were examined and imaged using a light microscope (Nikon, Tokyo, Japan).

### 2.6. Immunofluorescence Analysis

Paraffin-embedded sections were dewaxed and rehydrated through a graded ethanol series (100%, 95%, 80%, 70% ethanol, 5 min each), followed by antigen retrieval in 0.01 M citrate buffer (pH 6.0) by heating in a water bath (95–100 °C for 20 min). After cooling to room temperature naturally, sections were blocked with 5% bovine serum albumin (BSA) for 30 min to reduce non-specific binding, and then incubated overnight at 4 °C with primary antibodies against ZO-1, Occludin, and MUC2 (Abcam, Cambridge, UK, diluted 1:1000). The next day, sections were washed three times with PBS (pH 7.4) (5 min each) and then incubated with Cy3-conjugated secondary antibody (Beyotime Biotechnology, Shanghai, China, diluted 1:500) at room temperature for 1 h in the dark. Nuclei were stained with DAPI (1 μg/mL) for 5 min, and images were acquired using a fluorescence microscope (ECHO, Los Angeles, CA, USA) [18,23,24].

### 2.7. RT-qPCR Analysis

Total RNA was extracted from colon tissues with Trizol reagent (Shanghai Sangon, Shanghai, China), reverse-transcribed into cDNA using the PrimeScript™ RT kit with gDNA Eraser (Takara Bio, Beijing, China), and quantified by qPCR with TB Green^®^ Premix Ex Taq™ II (Takara, Beijing, China). Primer sequences are listed in Table S1.

### 2.8. Western Blot Analysis

Colon tissues were homogenized in RIPA buffer containing 1% PMSF. Protein concentrations were measured with a BCA kit. Proteins were separated by 10% SDS-PAGE, transferred to PVDF membranes, blocked with 5% skim milk, and incubated with primary antibodies (1:1000, 4 °C, overnight) and HRP-conjugated secondary antibodies (1 h, 37 °C). Band intensities were quantified using ImageJ 1.54f [25].

### 2.9. 16S rRNA Sequencing Analysis

The gut microbiota composition was analyzed by 16S rRNA gene sequencing, with both sequencing and bioinformatic processing conducted by Biomarker Technologies Co., Ltd. (Beijing, China). Genomic DNA was extracted using the TGuide S96 Magnetic Soil/Stool DNA Kit (Tiangen Biotech, Beijing, China) following the manufacturer’s protocol. The PCR products were purified, quantified, and normalized using standard procedures before being sequenced on the Illumina NovaSeq 6000 (Illumina, San Diego, CA, USA) platform.

### 2.10. Metabolomics Analysis

Metabolomic analysis was performed by Novogene Co., Ltd. (Beijing, China) based on established protocols. Briefly, intestinal tissue samples were ground into powder using liquid nitrogen, and total metabolites were extracted using a pre-chilled 80% methanol–water solution. After centrifugation at 12,000× *g* for 15 min at 4 °C, the supernatant was collected and filtered through a 0.22 μm membrane. The filtered samples were subjected to ultra-performance liquid chromatography–tandem mass spectrometry (UPLC-MS/MS) analysis using a Waters ACQUITY UPLC system coupled with a Xevo TQ-XS mass spectrometer (Waters, Milford, CT, USA). Chromatographic separation was performed on a Waters BEH C18 column (2.1 mm × 100 mm, 1.7 μm) at 40 °C. The mobile phase consisted of solvent A (0.1% formic acid in water) and solvent B (0.1% formic acid in acetonitrile) with a gradient elution program. The mass spectrometer was operated in both positive and negative ion modes. Data acquisition was carried out in multiple reaction monitoring (MRM) mode. Metabolite identification and quantification were performed using the MassLynx software (version 4.1) by matching with the Novogene self-built database.

### 2.11. Cell Culture and Viability

Caco-2 and RAW 264.7 cell cultures were established as previously described with minor modifications [26]. RAW 264.7 cells were cultured in Dulbecco’s Modified Eagle Medium (DMEM) supplemented with 10% (*v*/*v*) fetal bovine serum (FBS) and 1% penicillin-streptomycin. Caco-2 cells were maintained in DMEM containing 15% (*v*/*v*) FBS, 1% penicillin-streptomycin, and 1% non-essential amino acids. Both lines were incubated at 37 °C in a humidified 5% CO_2_ atmosphere.

CIPs’ effect on cells via the CCK-8 assay is as follows. Caco-2 cells were seeded in 96-well plates at 1 × 10^5^ cells/mL and cultured for 24 h. Cells were then treated with 100 μL CIP solutions (12.5–1000 μg/mL), DMEM as the control. After 24 h incubation, 10 μL CCK-8 solution was added, followed by 2 h incubation. Optical density was measured using a Multiskan Spectrum microplate reader.

In the 5-FU-induced Caco-2 barrier dysfunction model, cells were seeded at 1 × 10^6^ cells/mL in DMEM, pretreated with 50 μg/mL CIPs for 12 h, and then exposed to 5-FU for 24 h to induce functional impairment. The integrity of tight junctions was analyzed by immunofluorescence (Section 2.6) and fluorescence microscopy, while concurrent oxidative stress was evaluated by measuring ROS content following the previous method [27].

The LPS-induced RAW264.7 cell inflammation model involved seeding RAW264.7 cells at 1 × 10^6^ cells/mL in DMEM, with the blank control using DMEM. After the appropriate attachment period, cells were pretreated with 50 μg/mL CIPs for 12 h, followed by stimulation with 1 μg/mL LPS (Sigma, Virginia Beach, VA, USA) for 24 h. This LPS concentration induces pro-inflammatory cytokines without excessive cell death [28], and gene expression was analyzed via qPCR as described in Section 2.7.

### 2.12. Cell Polarization

In the 5-FU-induced polarization model using RAW 264.7 cells, the cells were seeded at a density of 1 × 10^6^ cells/mL in 24-well plates, with a blank control group cultured in DMEM. The cells were pretreated with 50 μg/mL CIPs for 12 h and subsequently exposed to 20 μg/mL 5-FU for 24 h. They were subsequently fixed with 4% paraformaldehyde, permeabilized with 0.1% Triton X-100, and blocked with 5% BSA. They were then incubated with primary antibodies against CD86 (1:200, Abcam, Cambridge, UK) and CD163 (1:200, Abcam, Cambridge, UK) at 4 °C overnight, followed by incubation with Cy3-conjugated secondary antibody (1:500, Beyotime) for 1 h at room temperature in the dark. Nuclei were counterstained with DAPI. Macrophage polarization was then assessed via fluorescence microscopy [29]. M1 macrophages were identified via the pro-inflammatory marker CD86, and M2 macrophages via the anti-inflammatory marker CD163.

### 2.13. Statistical Analysis

All data are presented as mean ± SEM. Data normality was examined by the Shapiro–Wilk test, and homogeneity of variance was verified using Levene’s test. Statistical analyses were performed with GraphPad Prism 10.1. One-way ANOVA followed by Dunnett’s post hoc test was applied for multiple group comparisons. Statistical significance was defined as *p* < 0.05, * *p* < 0.01, ** *p* < 0.001, and *** *p* < 0.0001. All experiments were conducted independently in triplicate.

## 3. Results

### 3.1. Characterization of CIPs

Previous studies have demonstrated the characterization of polysaccharides: the yield of CIPs was 3.13% (*w*/*w*), with 63.53 ± 1.59% total carbohydrate content, 6.04 ± 0.19% uronic acid content, and an average molecular weight of 3.97 kDa. Monosaccharide composition analysis revealed that CIPs consist of six monosaccharides, namely mannose (Man), rhamnose (Rha), glucuronic acid (GlcA), galacturonic acid (GalA), xylose (Xyl), and fucose (Fuc), with a molar ratio of 0.12:0.15:0.05:0.14:0.10:0.16. Additionally, CIPs have a sulfate content of 13.05 ± 0.75% (*w*/*w*) and a protein contamination of 4.63 ± 0.14% (*w*/*w*), as determined by the barium chloride–gelatin turbidimetric method and Coomassie Brilliant Blue assay, respectively [19]. Endotoxin testing via the Limulus Amebocyte Lysate (LAL) assay showed that the endotoxin content of CIPs was 0.002964 EU/mg, which is within the acceptable range for in vitro and in vivo biological activity evaluations.

### 3.2. Effects of CIPs on Clinical Parameters in Mice with 5-FU-Induced Intestinal Injury

Body weight, diarrhea scores, and hematochezia were monitored daily (Figure 2B); these are classic indicators of chemotherapy-related intestinal damage. These symptoms were notably alleviated with CIP treatment. Diarrhea scores further confirmed CIPs’ protective role, showing substantial symptom relief in contrast to the 5-FU group (Figure 2C). Moreover, colonic shortening, another hallmark of 5-FU-induced intestinal injury, was significantly mitigated by CIPs (Figure 2D,G). Altogether, these results demonstrate that CIPs effectively alleviate the clinical symptoms of 5-FU-induced intestinal toxicity, including weight loss, diarrhea, and colonic shortening.

Histopathological assessments provided further evidence for these results. H&E staining (Figure 2E,H) showed severe structural impairment in the 5-FU group: goblet cell loss, submucosal edema, crypt structural disruption, neutrophilic infiltration, and widespread ulceration. By comparison, CIP administration maintained intestinal structure and markedly alleviated tissue damage, with the high-dose group showing more pronounced protective effects. Furthermore, AB-PAS staining (Figure 2F,I) revealed that mucus-secreting goblet cells were markedly depleted in the 5-FU group, resulting in a significant reduction in the AB-positive area (*p* < 0.001). In contrast, CIP intervention robustly reversed this depletion, with both LCIP and HCIP treatments significantly restoring the AB-positive area to near-baseline levels, with the high-dose HCIP group exhibiting a more pronounced restoration of the intestinal mucus barrier.

### 3.3. Effects of CIPs on Colonic Tight Junction Protein Expression

**Figure 3:**
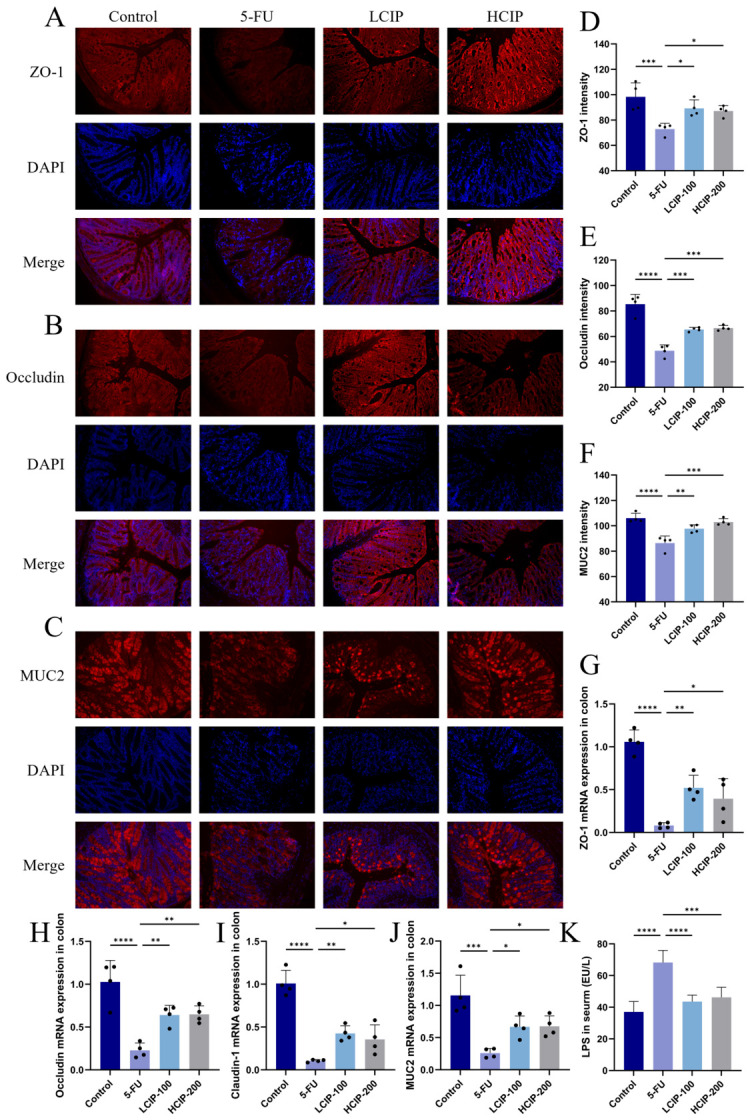
CIPs ameliorate 5-FU-induced intestinal injury by restoring intestinal barrier integrity. (**A**,**B**) Immunofluorescence staining of tight junction proteins (TJs: ZO-1 and Occludin) in colon tissues. (**C**) Immunofluorescence staining of MUC2 in colon tissues. (**D**–**F**) Quantitative analysis of immunofluorescence signal intensity for TJs and MUC2. (**G**–**J**) Relative mRNA expression levels of MUC2 and TJ-related genes (ZO-1, Occludin, Claudin-1) in colon tissues, determined by qPCR. (**K**) LPS content in serum. Data are presented as mean ± SEM (*n* = 4). * *p* < 0.05, ** *p* < 0.01, *** *p* < 0.001, and **** *p* < 0.0001 vs. the 5-FU group.

**Figure 4:**
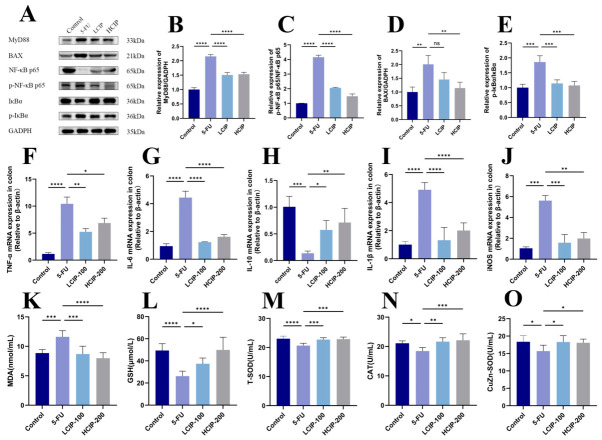
CIPs inhibit the NF-κB signaling pathway, inflammatory cytokines, and oxidative stress. (**A**) Protein expression levels of p65, p-p65, IκBα, p-IκBα, BAX, and MyD88 in colon tissues, with GADPH as the internal control. (**B**–**E**) Quantitative analysis of protein band intensities (*n* = 3). (**F**–**J**) Relative mRNA expression levels of TNF-α, IL-6, IL-10, IL-1β, and iNOS in colon tissues, determined by qPCR (*n* = 4). (**K**–**O**) Levels of MDA, GSH, SOD, and CAT in serum (*n* = 6). Data are presented as mean ± SEM. ns, not significant; * *p* < 0.05, ** *p* < 0.01, *** *p* < 0.001, and **** *p* < 0.0001 vs. the 5-FU group.

5-FU exposure markedly reduced protein levels of ZO-1, Occludin, and MUC2 in colon tissue (Figure 3A–C), suggesting compromised intestinal barrier integrity. These decreases were largely reversed by CIP treatment: CIP administration notably increased Occludin, ZO-1, and MUC2 expression in the colon of the 5-FU group, bringing these key proteins closer to control levels (Figure 4A,D–F). Meanwhile, CIPs also effectively suppressed the 5-FU-induced elevation of serum LPS, reduced LPS translocation, and ameliorated 5-FU-induced intestinal barrier injury (Figure 3K). These outcomes show that CIPs effectively ease 5-FU-induced impairment of gut barrier function through boosting critical tight junction protein expression.

The mRNA expression levels of MUC2, ZO-1, Occludin, and Claudin-1 were markedly suppressed in the 5-FU group (Figure 4G–I). This included the gene for the mucin MUC2 and the tight junction proteins ZO-1, Occludin, and Claudin-1. Importantly, CIP treatment markedly raised mRNA expression of these proteins. In line with immunofluorescence results, these PCR data strongly suggest that CIPs reduce 5-FU-induced gut damage by reestablishing mRNA expression of TJs and MUC2, thus aiding intestinal barrier recovery.

### 3.4. Effects of CIPs on NF-κB Signaling Pathway and Inflammatory Cytokines

As a central signaling axis in inflammation and immunity, the MyD88/NF-κB pathway is indispensable for host defense. 5-FU exposure activated this pathway, shown by elevated MyD88 levels and higher phosphorylation ratios of p-p65/p65 and p-IκBα/IκBα in colon tissues (Figure 4A–E). CIP treatment blocked this activation by lowering MyD88 protein levels and suppressing nuclear translocation of phosphorylated IκBα and NF-κB p65. These findings suggest that CIPs may alleviate 5-FU-induced intestinal inflammation in association with the regulation of the MyD88/NF-κB pathway.

**Figure 5:**
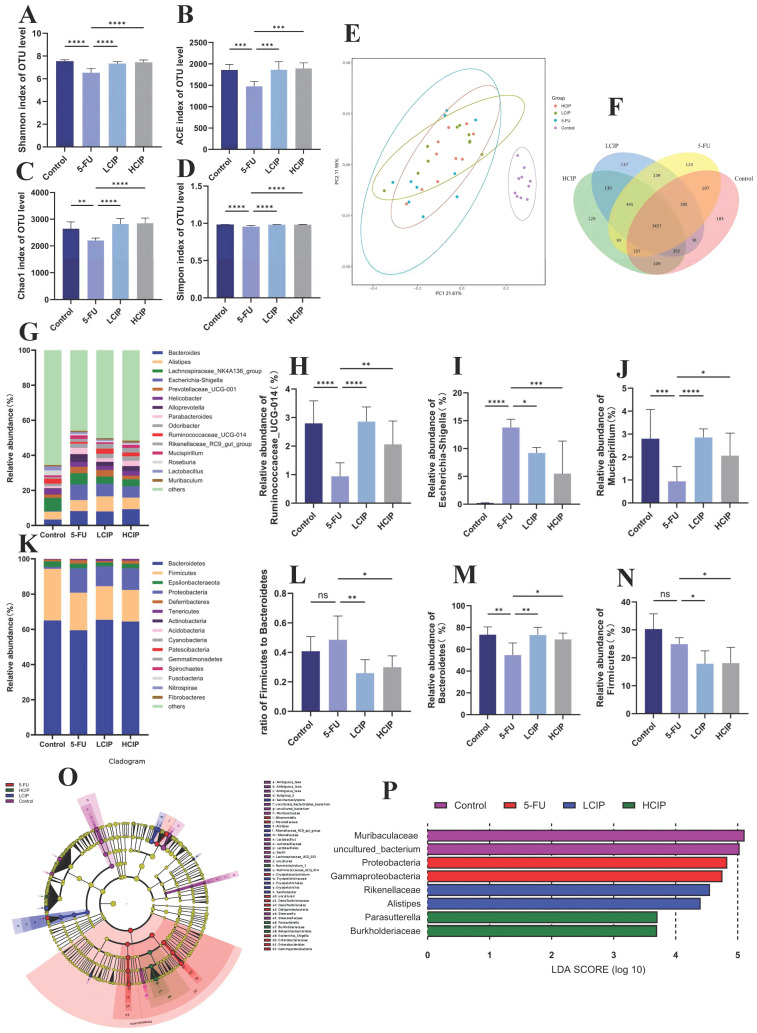
CIPs regulate gut microbiota composition in 5-FU-treated mice. (**A**) Shannon index. (**B**) ACE index. (**C**) Chao1 index. (**D**) Simpson index. (**E**) Principal coordinate analysis (PCoA) based on weighted UniFrac distances. (**F**) Venn diagram analysis of operational taxonomic units (OTUs). (**G**) Genus-level composition of gut microbiota. (**H**–**J**) Relative abundance of Ruminococcaceae_UCG-014 (**H**), Escherichia-Shigella (**I**), and Mucispirillum (**J**). (**K**) Phylum-level composition of gut microbiota. (**L**) *Firmicutes*/*Bacteroidota (F/B)* ratio. (**M**,**N**) Relative abundance of *Bacteroidota* (**M**) and *Firmicutes* (**N**). (**O**) LEfSe plots highlighting taxa with the greatest differential abundance among groups. (**P**) Linear discriminant analysis (LDA), showing statistically significant differences in microbial taxa across groups. Data are presented as mean ± SEM (*n* = 6). ns, not significant; * *p* < 0.05, ** *p* < 0.01, *** *p* < 0.001, **** *p* < 0.001 vs. 5-FU group.

In parallel, colon levels of pro-inflammatory factors IL-1β, IL-6, and TNF-α were significantly higher, whereas IL-10 was notably lower than controls, aligning with their function in driving mucosal injury and systemic inflammation. CIP intervention rebalanced these factors by reducing pro-inflammatory factor expression and increasing IL-10 in the colon, further supporting their anti-inflammatory effects (Figure 5F–I).

Oxidative stress is closely connected to inflammation. The upregulation of inducible nitric oxide synthase (iNOS) serves as the primary mediator of the excessive generation of nitric oxide. Colonic iNOS content was dramatically increased in 5-FU-exposed tissues but significantly mitigated by CIP treatment (Figure 5J), suggesting preserved antioxidant capacity. Reduced colonic iNOS levels induced by CIPs were associated with alleviated tissue damage, supporting the well-documented link between iNOS suppression and reduced injury severity. Collectively, these results indicate that CIPs ameliorate intestinal injury resulting from 5-FU treatment by blunting NF-κB signaling and attenuating the downstream inflammatory cytokine response.

### 3.5. Effects of CIPs on Serum Oxidative Stress Indexes

Disruption of redox homeostasis represents a hallmark of intestinal injury subsequent to 5-FU administration. This intestinal injury is characterized by increased lipid peroxidation and compromised antioxidant defense. Relative to the control group, serum malondialdehyde (MDA) was significantly elevated in the 5-FU group (Figure 4K), indicating increased oxidative damage. Conversely, 5-FU exposure reduced serum antioxidant indicators: glutathione (GSH), catalase (CAT), and superoxide dismutase (SOD) were significantly decreased compared with controls (Figure 4L–O). Notably, CIP treatment reversed these alterations: serum MDA levels were notably diminished in the 5-FU + CIPs group, reflecting mitigated lipid peroxidation. Meanwhile, CIPs upregulated serum GSH and restored CAT and SOD activities, indicating enhanced endogenous antioxidant capacity. These results demonstrate that CIPs alleviate 5-FU-induced systemic oxidative damage both by diminishing markers of lipid peroxidation and potentiating cellular antioxidant enzymes.

### 3.6. Effects of CIPs on Gut Microbiota Composition

**Figure 6:**
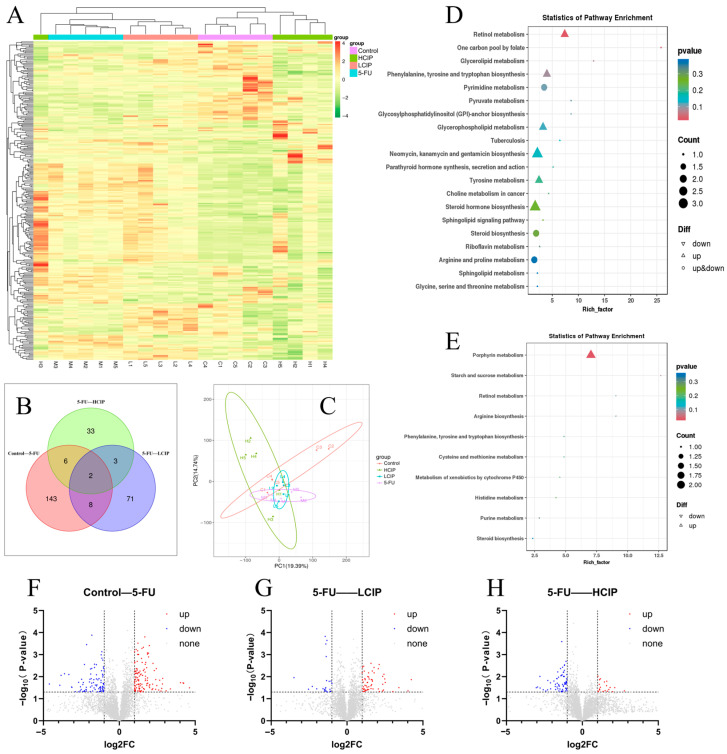
CIPs alleviate 5-FU-induced intestinal injury in mice by regulating metabolic pathways. (**A**) Heatmap of differential metabolite clustering in the control, as well as 5-FU-, LCIP-, and HCIP-treated mice. (**B**) Venn diagram analysis of differential metabolites among groups. (**C**) PCA of metabolites in the control, as well as 5-FU-, LCIP-, and HCIP-treated mice. (**D**) KEGG enrichment bubble plot of differential metabolites between the control and 5-FU groups. (**E**) KEGG enrichment bubble plot of differential metabolites between the 5-FU and LCIP groups. (**F**) Volcano plot of differentially expressed genes between the control and 5-FU mice. (**G**) Volcano plot of differentially expressed genes between the 5-FU- and LCIP-treated mice. (**H**) Volcano plot of differentially expressed genes between the 5-FU- and HCIP-treated mice.

Alpha diversity indices (Shannon, Ace, Chao, and Simpson) were significantly lower in the 5-FU group than in the control group (Figure 5A–D). In contrast, CIP administration increased these indices dose-dependently, suggesting recovered microbial abundance and diversity. A Venn diagram demonstrated specific operational taxonomic units (OTUs) numbered 183 in the control group, 124 in the 5-FU group, and 137 in the low-dose CIP group, respectively (Figure 6F), confirming that CIPs restored 5-FU-induced gut microbiota dysbiosis.

Relative to controls, 5-FU-induced mice showed significant depletion of *Bacteroidota* (control: 73.35 ± 7.17%; 5-FU: 54.75 ± 11.08%, *p* < 0.01; Figure 5M) and *Firmicutes* (control: 31.07 ± 5.55%; 5-FU: 25.14 ± 2.40%, * *p* < 0.05). Due to a larger reduction amplitude of *Bacteroidota* (25.36%) than *Firmicutes* (19.08%), the *Firmicutes*/*Bacteroidota* (*F*/*B*) ratio was significantly elevated (control: 0.425; 5-FU: 0.464, *p* < 0.05; Figure 5H); 5-FU also perturbed taxa like Campylobacter, exacerbating dysbiosis. CIPs reversed these abnormalities: they restored *Bacteroidota* to near-control levels (LCIP: 73.11 ± 6.89%; HCIP: 69.15 ± 7.32%), downregulated Proteobacteria (Figure 5N), and further reduced *Firmicutes* (LCIP: 16.79 ± 3.76%; HCIP: 16.99 ± 5.85%), normalizing the *F/B* ratio to 0.245–0.262. This confirms that CIPs alleviate 5-FU-induced intestinal injury by balancing the *F*/*B* ratio and reshaping gut microbiota.

At the genus level, CIPs regulated 5-FU-induced changes in *Ruminococcaceae_UCG-014*. The 5-FU group showed notably elevated *Escherichia-Shigella* and altered *Mucispirillum* abundance, which were effectively restored by CIP administration (Figure 5G–J). LEfSe analysis (Figure 5N) identified bacterial taxa with inter-group enrichment differences, with linear discriminant analysis (LDA)

### 3.7. Effects of CIPs on Fecal Metabolites

Untargeted metabolomic analysis via UPLC-MS explored CIPs’ metabolic regulatory effects in 5-FU-induced intestinal injury. Distinct clustering patterns were observed via principal component analysis, indicating a discernible separation between the microbial communities of the 5-FU and CIPs-treated cohorts, indicating distinct metabolic profile alterations by CIP intervention (Figure 6B,C) Differential metabolites regulated by CIPs were significantly enriched in four key pathways: retinol metabolism, arginine metabolism, phenylalanine–tyrosine–tryptophan metabolism, and steroid biosynthesis (Figure 6D,E). These pathways are closely associated with nutrient metabolism, immune regulation, and redox balance—critical for maintaining intestinal homeostasis. Enrichment of these pathways suggests CIPs alleviate 5-FU-induced metabolic disorders by modulating key metabolic pathways, contributing to metabolic balance restoration.

### 3.8. Effects of CIPs on Cellular Inflammation, Oxidative Damage, and Intestinal Barrier Function In Vitro

**Figure 7:**
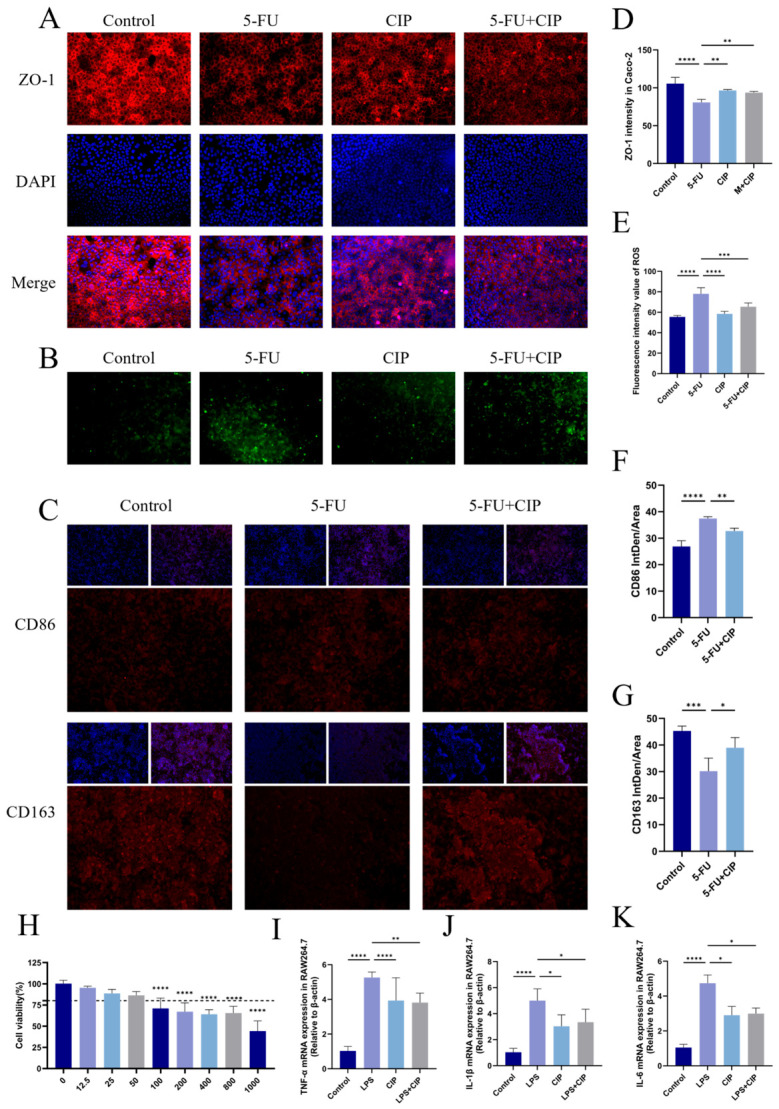
CIPs inhibit cellular inflammation and oxidative damage, induce macrophage M2 polarization, and improve intestinal barrier function. (**A**) Immunofluorescence analysis of ZO-1 expression in Caco-2 cells after CIP treatment. (**B**) Fluorescence images of ROS in Caco-2 cells after CIP treatment. (**C**) Immunofluorescence analysis of RAW264.7 cell polarization regulated by CIPs. (**D**) Quantitative analysis of ZO-1 fluorescence intensity. (**E**) Fluorescence intensity value of intracellular ROS. (**F**,**G**) Quantitative analysis of RAW264.7 cell polarization regulated by CIPs. (**H**) Cytotoxicity of CIPs at different concentrations on Caco-2 cells, determined by CCK-8 assay. (**I**–**K**) Relative mRNA expression levels of TNF-α (**I**), IL-1β (**J**), and IL-6 (**K**) in RAW264.7 cells after CIP treatment (model group: LPS-treated group). Data are presented as mean ± SEM (*n* = 4). * *p* < 0.05, ** *p* < 0.01, *** *p* < 0.001, and **** *p* < 0.0001 vs. the LPS/model group.

In vitro experiments demonstrated multifaceted protective effects of CIPs in cellular inflammation models. At 12.5–50 μg/mL, CIPs showed no cytotoxicity toward intestinal-like Caco-2 cells. In LPS-stimulated cells, CIP intervention attenuated the upregulation of pro-inflammatory mediators IL-6 and TNF-α mRNA, while increasing the expression of anti-inflammatory IL-10 (Figure 7I–K). Additionally, CIPs decreased intracellular ROS production (Figure 7B), mitigating LPS-induced inflammation and oxidative stress. Furthermore, CIPs alleviated 5-FU-mediated intestinal barrier disruption, as evidenced by restored ZO-1 expression (Figure 7A). Collectively, these findings indicate that CIPs inhibit cellular inflammatory responses and oxidative damage while improving intestinal barrier function in vitro.

### 3.9. Effects of CIPs on Macrophage Polarization In Vitro

To explore CIPs’ role in regulating macrophage polarization, RAW264.7 cells were treated with 5-FU to elicit M1 polarization, followed by CIP intervention to evaluate M2 polarization promotion. Immunofluorescence showed that 5-FU treatment significantly increased M1 macrophage marker fluorescence intensity, indicating effective M1 induction. In contrast, CIP treatment reduced M1-polarized macrophage proportion, reflecting a shift toward M2—consistent with inflammatory cytokine mRNA expression trends. CIPs significantly alleviated cellular inflammation and promoted macrophage polarization toward M2, suppressing the secretion of pro-inflammatory cytokines while enhancing the transcription of anti-inflammatory genes.

## 4. Discussion

5-FU is a widely used chemotherapeutic agent for solid tumors, but its clinical application is severely limited by intestinal toxicity. Natural polysaccharides with multi-target regulatory activities and good biocompatibility are promising candidates for alleviating chemotherapy-induced intestinal injury. This study systematically investigated the protective effects and mechanisms of CIPs against 5-FU-induced intestinal injury, providing mechanistic evidence for its potential as a natural adjuvant therapy.

Weight loss, diarrhea, and colonic shortening are classical phenotypic indicators for assessing the severity of 5-FU-induced intestinal mucositis in preclinical animal models [3]. The prominent ameliorative effects of CIPs on these clinical abnormalities are comparable to those of other natural polysaccharides documented in recent studies, supporting the potential of CIPs as a promising intervention for chemotherapy-related intestinal discomfort. The observed histological improvement further indicates that CIPs can effectively preserve colonic tissue structure, which is essential for restoring normal intestinal physiological function. Moreover, AB-PAS staining revealed that CIPs restored goblet cell numbers and mucin secretion, which serves a central function in safeguarding the intestinal epithelium from pathogenic invaders by sustaining the mucus barrier [30,31]. These results are in line with prior work that supports the concept that natural polysaccharides preserve mucosal integrity [32,33] and highlight fish-derived swim bladder polysaccharides as a novel option for chemotherapy-induced mucositis.

The intestinal epithelial barrier (tight junctions and mucus layer) serves as the primary defense against intestinal damage. 5-FU disrupts tight junctions and mucin secretion, thereby increasing intestinal permeability and aggravating mucosal injury. In our study, CIP intervention markedly upregulated the expression of both TJs and MUC2 at the mRNA level, as evidenced by both transcriptional and translational analyses, which contributed to the recovery of intestinal epithelial integrity. These results, consistent with barrier-protective effects for astragalus polysaccharides [28], indicate that maintaining TJ integrity and mucus secretion constitutes a key mechanism underlying the intestinal protective effects of CIPs.

Given the close interplay between barrier dysfunction and intestinal inflammation, we next investigated inflammatory signaling pathways. Consistent with earlier studies [34], 5-FU exposure was associated with activation of the MyD88/NF-κB pathway, along with increased p-IκBα and NF-κB p65; these changes coincided with a “cytokine storm” characterized by increased TNF-α, IL-6, and IL-1β and decreased IL-10. CIP intervention was accompanied by the suppression of this activation, downregulation of MyD88, inhibition of IκBα, and NF-κB p65 phosphorylation. These findings align with evidence that polysaccharides attenuate intestinal inflammation via NF-κB inhibition [35,36,37], suggesting a potential correlation between CIPs’ protective effects against 5-FU-induced mucositis and their modulation of MyD88-dependent NF-κB signaling. Notably, the current observations are based on changes in protein expression and phosphorylation.

Oxidative damage acts as a core pathological driver in 5-FU-triggered intestinal injury [21]. The regulatory effects of CIPs on serum oxidative stress indexes reflect their robust antioxidant capacity, which is consistent with the reported bioactivity of fish swim bladder-derived polysaccharides. This antioxidant effect helps interrupt the vicious cycle between oxidative stress and intestinal inflammation, representing a key mechanism underlying the intestinal protection of CIPs. In addition, CIPs downregulated colonic iNOS expression, further mitigating oxidative damage [38,39]. These results are regarded as the antioxidant properties previously reported for swim bladder polysaccharides and confirm oxidative stress mitigation as another key protective mechanism of CIPs.

Gut microbiota dysbiosis is a well-established factor that aggravates 5-FU-induced intestinal toxicity [10,34]. The regulatory effects of CIPs on microbial diversity, phylum composition, and *F/B* ratio align with the prebiotic properties of most natural polysaccharides. Notably, the preferential enrichment of *Bacteroidota* by CIPs is a distinctive feature of this marine polysaccharide, as this phylum is the major producer of short-chain fatty acids that maintain intestinal homeostasis [40,41]. Notably, CIP intervention reversed these changes in a dose-dependent manner, boosting microbial diversity, enriching beneficial *Bacteroidota*, lowering *Proteobacteria* levels, and normalizing the *F/B* ratio. At the genus level, CIPs exerted a regulatory effect on the gut microbiota by not only reducing pro-inflammatory taxa like Escherichia-Shigella but also revitalizing beneficial genera responsible for SCFA production. Consistent with prebiotic properties of natural polysaccharides [42,43,44], CIPs attenuate intestinal injury via microbial community restoration and a concurrent expansion of beneficial bacteria.

Metabolic dysregulation increasingly contributes to chemotherapy-induced intestinal injury, as perturbed nutrient metabolism and metabolite imbalance exacerbate inflammation, barrier dysfunction, and microbial dysbiosis [45]. Our untargeted metabolomic analysis showed CIPs significantly altered the metabolic profile of 5-FU-treated mice, with differential metabolites enriched in retinol metabolism, arginine metabolism, phenylalanine–tyrosine–tryptophan metabolism, and steroid biosynthesis—highlighting metabolic modulation as an important supplementary mechanism [46,47,48].

Macrophages play a dual role in intestinal injury: M1 macrophages promote inflammation, whereas M2 macrophages facilitate anti-inflammatory responses and tissue repair [49,50]. In vitro experiments showed 5-FU induced M1 polarization of RAW264.7 macrophages (increased M1 markers). CIP intervention inhibited M1 polarization, promoted M2 polarization, lowered the classical pro-inflammatory mediators (IL-1β, IL-6, and TNF-α), and elevated the anti-inflammatory factor IL-10—consistent with in vivo reduced intestinal inflammation. This supports macrophage polarization as a critical target of CIPs’ anti-inflammatory activities, extending their immunomodulatory properties to macrophage function and providing novel mechanistic insights [51]. The innovation of this study is that it pioneers the application of CIPs in the intervention of chemotherapy-induced intestinal injury and reveals a new multi-target mechanism integrating barrier protection, immune regulation, microbial remodeling, and metabolic modulation, which is distinct from the single regulatory mechanism of most reported polysaccharides. The limitation is that this study is limited to animal experiments, and the structural basis of CIP activity and clinical application potential need to be further explored.

## 5. Conclusions

This study demonstrates that CIPs protect against 5-FU-induced intestinal injury by enhancing barrier integrity, suppressing inflammation, alleviating oxidative stress, reshaping gut microbiota, and modulating metabolism. In vitro, CIPs further promoted anti-inflammatory macrophage polarization, supporting their immunomodulatory role. These findings highlight CIPs as a promising marine-derived natural adjuvant to improve chemotherapy tolerance and promote sustainable utilization of marine resources.

## Data Availability

The original contributions presented in this study are included in the article/Supplementary Materials. Further inquiries can be directed to the corresponding authors.

## Supplementary Materials

The following supporting information can be downloaded at: https://www.mdpi.com/article/10.3390/foods15081425/s1. Figure S1: CIPs modulate the concentrations of various fatty acids. Concentrations of fatty acids in samples analyzed by GC-MS (*n* = 5). (A) Acetic acid; (B) 3-Ethoxy-3-oxopropanoic acid; (C) 4-(4-Methylcyclohexyl-4)-oxobutanoic acid; (D) Butanoic acid; (E) 2-Methylbutyrate; (F) 2-n-Propyl-4-oxopentanoic acid; (G) Hexanoic acid; (H) Caproic acid. Data are presented as mean ± SEM. * *p* < 0.05, *** *p* < 0.001, ns: no significance vs. 5-FU group; Figure S2: CIPs regulate the levels of tryptophan and arginine metabolites. Concentrations of metabolites in samples analyzed by metabolomics (*n* = 5). (A) L-Formylkynurenine; (B) N-Acetyl-DL-tryptophan; (C) L-Arginine; (D) L-Arginine phosphate. Data are presented as mean ± SEM. * *p* < 0.05, ** *p* < 0.01, *** *p* < 0.001, ns: no significance vs. 5-FU group; Figure S3: Differential metabolite clustering heatmap Control_ Vs_ 5-FU; Figure S4: Differential metabolite clustering heatmap 5-FU _Vs_ HCIP; Figure S5: Differential metabolite clustering heatmap 5-FU _Vs_ LCIP; Table S1: Primer sequences used for RT-PCR experiments; Table S2: Diarrhea scoring system.
