# Supplementary material for: Larimichthys crocea Swim Bladder Polysaccharides Attenuate 5-Fluorouracil-Induced Intestinal Injury by Modulating the Gut–Metabolic Axis
Source: Foods. 2026 Apr 19;15(8):1425. doi: 10.3390/foods15081425 (PMC13116390; doi:10.3390/foods15081425)
Supplement: Supplementary file 1 [file foods-15-01425-s001.zip › foods-4235639-supplementary.pdf]

Table S1 Primer sequences used for RT-PCR experiments.

Genes	Forward primer	Reverse primer
β-actin	TCAGCAAGCAGGAGTAC	AACGCAGCTCAGTAACA
MUC2	GGTCCAGGGTCTGGATC	GCTCAGCTCACTGCCAT
ZO-1	GACCAATAGCTGATGTTG	TATGAAGGCGAATGATG
Occludin	GGCAAGCGATCATACCC	AGGCTGCCTGAAGTCAT
Claudin-1	AGTGCATGAGGTGCCTG	TGGCCACTAATGTCGCC
TNF-α	GCGACGTGGAACTGGCAGAAG	GCCACAAGCAGGAATGAGAAGAGG
IL-1β	TCGCAGCAGCACATCAACAAGAG	TGCTCATGTCCTCATCCTGGAAGG
IL-6	TGGAAATGAGAAAAGAGTTGTGC	CCAGTTTGGTAGCATCCATCA
IL-10	GAGGATCAGCAGGGGCCAGTAC	AAGGCAGTCCGCAGCTCTAGG
iNOS	ATGTCCGAAGCAAACAT	TAATGTCCAGGAAGTAG

Table S2 Diarrhea scoring system

Score	Fecal state
0	Well-formed pellets
1	Slightly wet and Soft stools
2	Loose and semi-formed stools
3	Watery stools

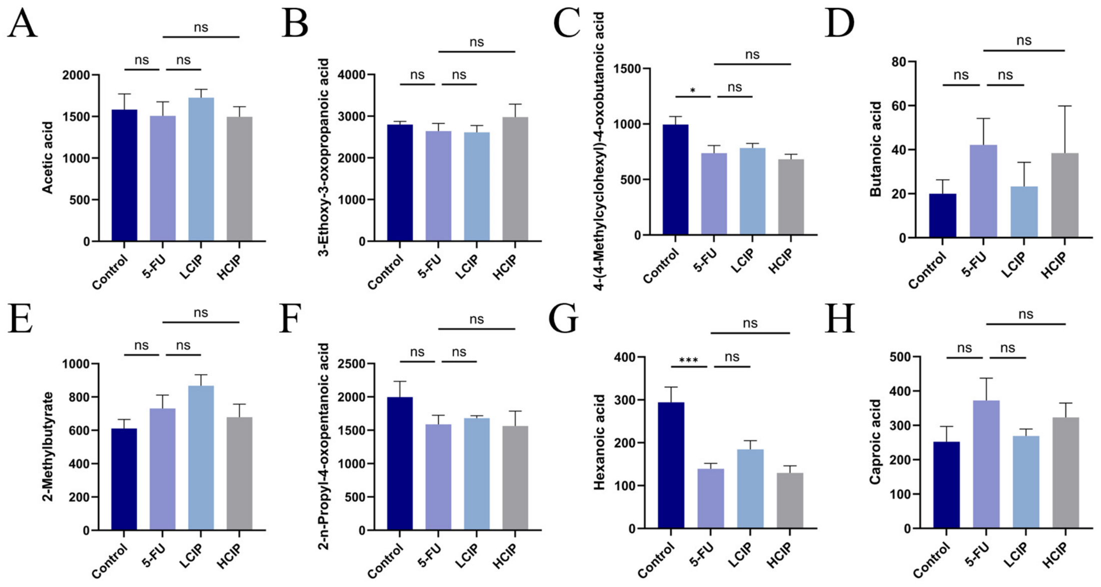

Fig. S1 CIPs modulate the concentrations of various fatty acids. Concentrations of fatty acids in samples analyzed by GC-MS (n=5). (A) Acetic acid; (B) 3-Ethoxy-3-oxopropanoic acid; (C) 4-(4-Methylcyclohexyl-4)-oxobutanoic acid; (D) Butanoic acid; (E) 2-Methylbutyrate; (F) 2-n-Propyl-4-oxopentanoic acid; (G) Hexanoic acid; (H) Caproic acid. Data are presented as mean ± SEM. *p < 0.05, ***p < 0.001, ns: no significance vs. 5-FU group.

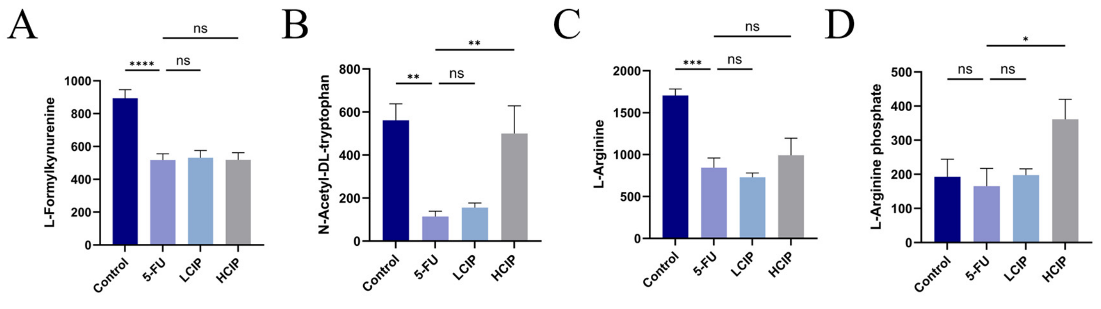

Fig. S2 CIPs regulate the levels of tryptophan and arginine metabolites. Concentrations of metabolites in samples analyzed by metabolomics (n=5). (A) L-Formylkynurenine; (B) N-Acetyl-DL-tryptophan; (C) L-Arginine; (D) L-Arginine phosphate. Data are presented as mean ± SEM. *p < 0.05, **p < 0.01, ***p < 0.001, ns: no significance vs. 5-FU group.

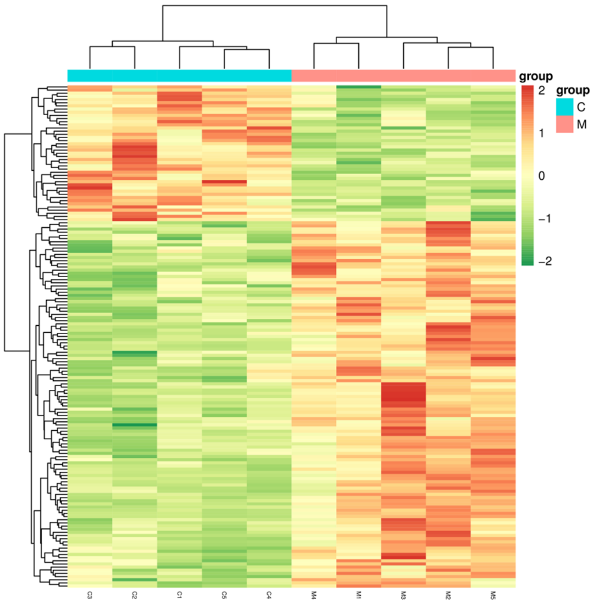

Fig. S3 Differential metabolite clustering heatmap   Control_ Vs_ 5-FU

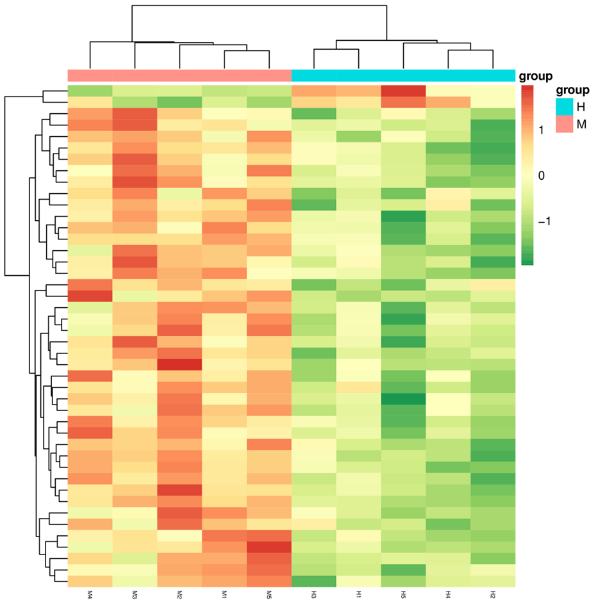

Fig. S4 Differential metabolite clustering heatmap   5-FU _Vs_ HCIP

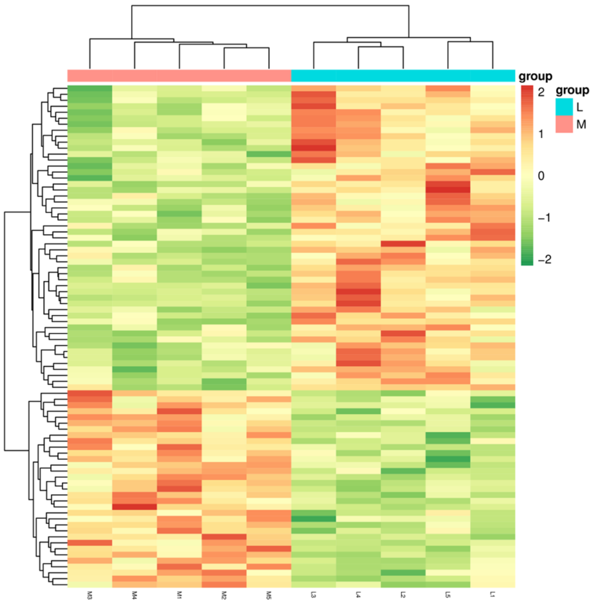

Fig. S5 Differential metabolite clustering heatmap 5-FU _Vs_ LCIP
